# Supplementary material for: In Vitro Digestion of Chestnut and Quebracho Tannin Extracts: Antimicrobial Effect, Antioxidant Capacity and Cytomodulatory Activity in Swine Intestinal IPEC-J2 Cells
Source: Animals (Basel). 2020 Jan 23;10(2):195. doi: 10.3390/ani10020195 (PMC7070574; doi:10.3390/ani10020195)
Supplement: Supplementary file 1 [file animals-10-00195-s001.pdf]

Supplementary Materials:

PCR conditions and gel electrophoresis

Briefly, DNA was isolated and purified from *E. coli* using the alkaline lysis method [1,2] The PCR primers were chosen from published sequences (Table S1). For F18+ screening, we amplified a fragment of FedF adhesin (more conserved in all antigenic variants); for F4+ screening we amplified the major fimbrial subunit FaeG. The primers were selected in order to have similar melting temperatures which resulted in a product of different sizes that could easily be identified in agarose gel.

Table S1. Primer sequences used in the PCR system for identification of *E. coli*. The primers were designed and checked using Vector NTI software (Thermo Fisher Scientific).

	Primer sequences (5′-3′)	Target gene	Size (bp)	Acc. Number	Melting Temperature (°C)
F4 (K88)-F<br>F4 (K88)-R	TGGATGACTGGTGATTTCAATGGTTCGG<br>TTAGTAATAAGTTATTGCTACGTTCAGCG	faeG	792	AY437806	68.7<br>64.8
F18-F<br>F18-R	ATTCTAGTGCGAGTAGTGCTCAAGTC<br>TTTTGCAATCGCAGGAACATAGATTGAAG	fedF	453	DQ914286	66.3<br>66.2

Each test was performed in a volume of 25 µl containing the following PCR components: dNTP (200 µM), PCR buffer, MgCl2 (2 mM), each primer set (10 pmol), 1,25U of pfu DNA polymerase (Promega), and DNA template (50 ng). The negative control consisted of 2 µl of water instead of DNA.

PCR amplifications were performed in a thermal cycle (T100 Thermal cycler, BIORAD) under the following conditions: an initial DNA denaturation step at 95 °C for 2 min, followed by 30 cycles beginning with 1 min of denaturation at 95 °C, 1 min for primer annealing at 57 °C, and 2 min of extension at 72 °C. The final extension step was performed at 72 °C for 5 min. The amplified PCR products were analyzed by electrophoresis in agarose gel after staining with Nancy stain (Sigma Aldrich) and visualization in a UV transilluminator (Figure S1).

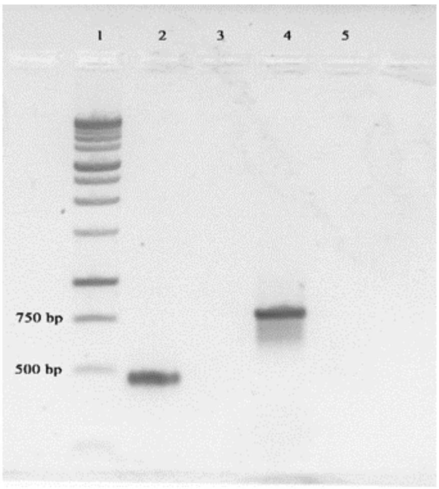

Figure 1. Gel electrophoresis of amplified PCR products.

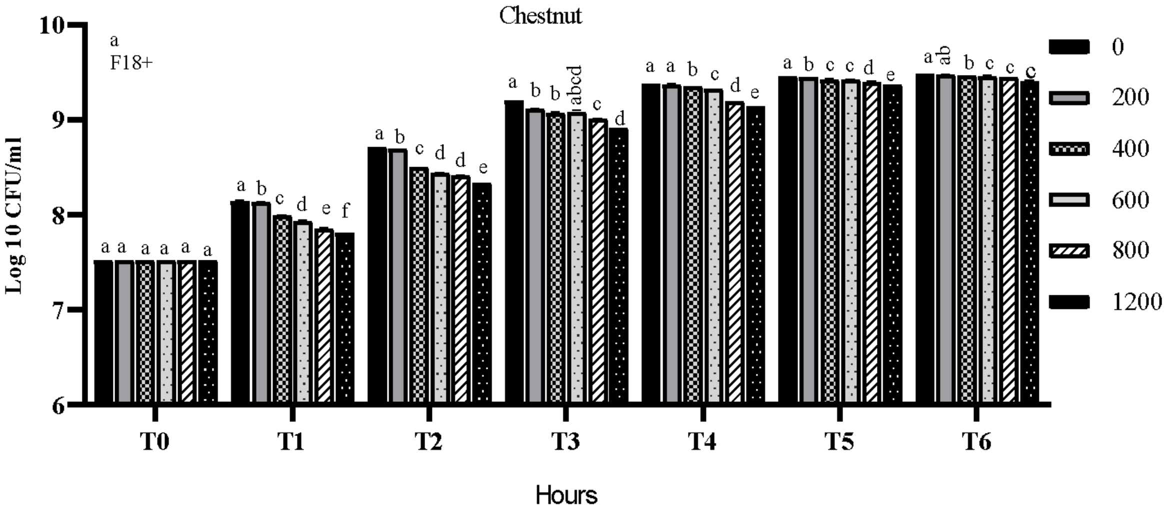

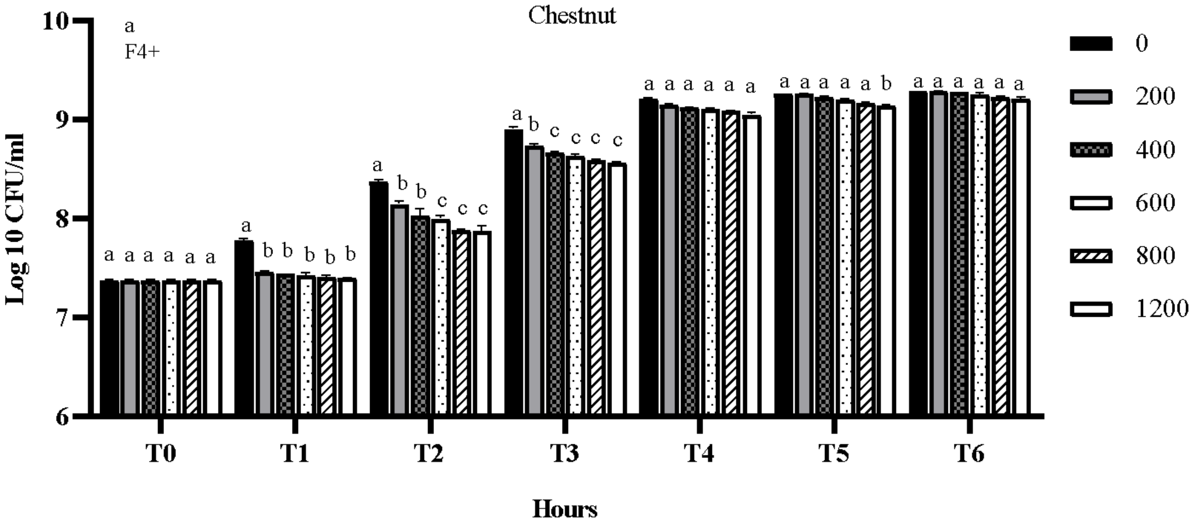

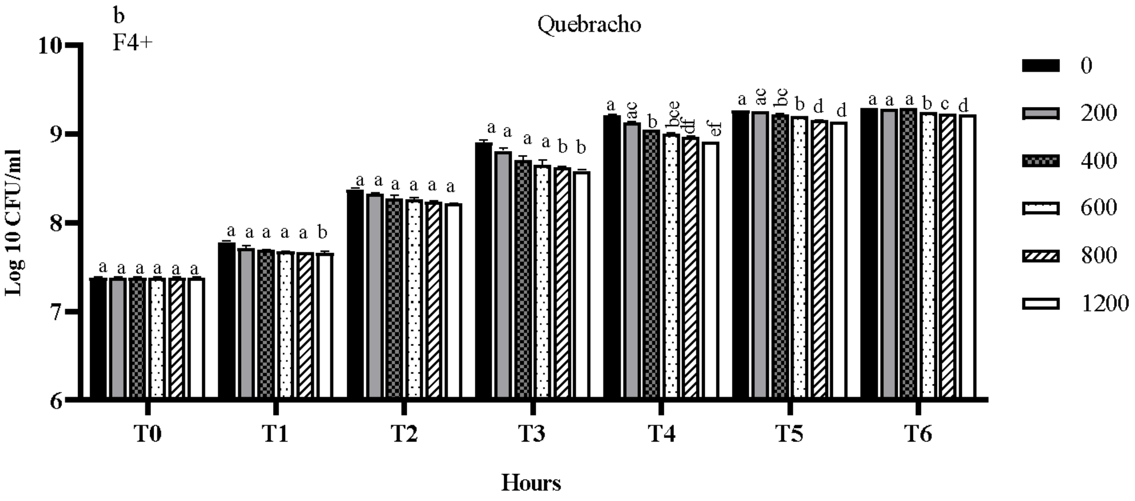

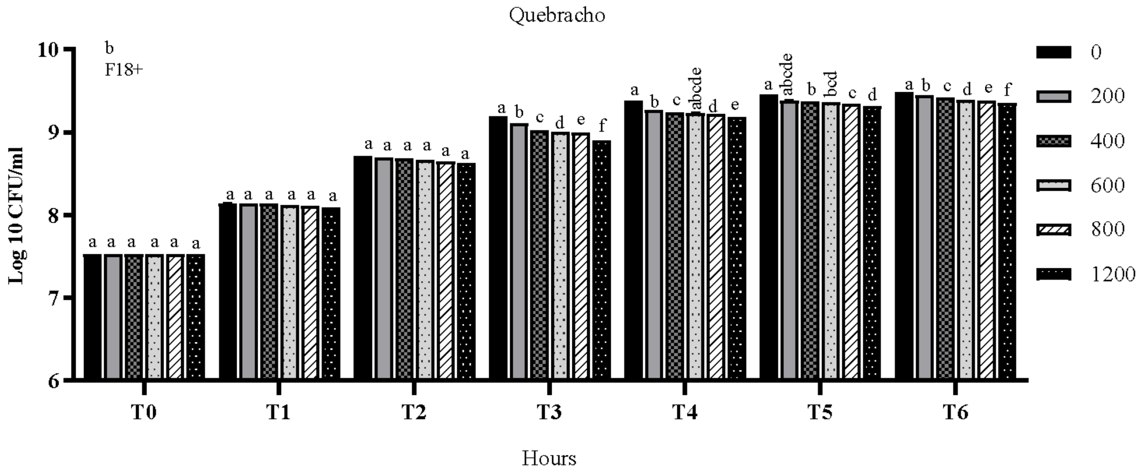

Figure 2. Effect of 0–1200 μg/mL of Ch (a) and Qu (b) tannins on *E. coli* F4+ and F18+ growth over time (T). Data are expressed as log10 CFU/mL lsmeans ± S.E.M. (n = 3). Different superscripts indicate significant differences at $p<0.05$ among different concentrations within the same time point.

References

1. Takaiwa, F. Increasing the production yield of recombinant protein in transgenic seeds by expanding the deposition space within the intracellular compartment. *Bioengineered* **2013**, *4*, 136–139.
2. Sambrook, J. *Russell Molecular Cloning: A Laboratory Manual*, 3rd ed.; CSHL: New York, NY, USA, 2001.
